# Actions of Rab27B‐GTPase on mammalian central excitatory synaptic transmission

**DOI:** 10.14814/phy2.14428

**Published:** 2020-05-01

**Authors:** Erwin R. Arias‐Hervert, Nicole Xu, Meredith Njus, Geoff G. Murphy, Yanan Hou, John A. Williams, Stephen I. Lentz, Stephen A. Ernst, Edward L. Stuenkel

**Affiliations:** ^1^ Molecular & Integrative Physiology Department University of Michigan Ann Arbor MI USA; ^2^ Michigan Neuroscience Institute University of Michigan Ann Arbor MI USA; ^3^ Internal Medicine University of Michigan Ann Arbor MI USA; ^4^ Cell & Developmental Biology University of Michigan Ann Arbor MI USA

**Keywords:** neurotransmission, RabGTPase, synapse

## Abstract

Members of the Rab3 gene family are considered central to membrane trafficking of synaptic vesicles at mammalian central excitatory synapses. Recent evidence, however, indicates that the Rab27B‐GTPase, which is highly homologous to the Rab3 family, is also enriched on SV membranes and co‐localize with Rab3A and Synaptotagmin at presynaptic terminals. While functional roles of Rab3A have been well‐established, little functional information exists on the role of Rab27B in synaptic transmission. Here we report on functional effects of Rab27B at SC‐CA1 and DG‐MF hippocampal synapses. The data establish distinct functional actions of Rab27B and demonstrate functions of Rab27B that differ between SC‐CA1 and DG‐MF synapses. Rab27B knockout reduced frequency facilitation compared to wild‐type (WT) controls at the DG/MF‐CA3 synaptic region, while increasing facilitation at the SC‐CA1 synaptic region. Remarkably, Rab27B KO resulted in a complete elimination of LTP at the MF‐CA3 synapse with no effect at the SC‐CA1 synapse. These actions are similar to those previously reported for Rab3A KO. Specificity of action on LTP to Rab27B was confirmed as LTP was rescued in response to lentiviral infection and expression of human Rab27B, but not to GFP, in the DG in the Rab27B KO mice. Notably, the effect of Rab27B KO on MF‐CA3 LTP occurred in spite of continued expression of Rab3A in the Rab27B KO. Overall, the results provide a novel perspective in suggesting that Rab27B and Rab3A act synergistically, perhaps via sequential effector recruitment or signaling for presynaptic LTP expression in this hippocampal synaptic region.

## Graphical abstract

Members of the Rab3 gene family are considered central to membrane trafficking of synaptic vesicles at mammalian central excitatory synapses. While functional roles of Rab3A have been well‐established, little functional information exists on the role of Rab27B in synaptic transmission. Our results provide a novel perspective in suggesting that Rab27B and Rab3A act synergistically, perhaps via sequential effector recruitment or signaling for presynaptic LTP expression in this hippocampal synaptic region.

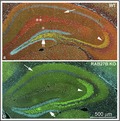

## 1 INTRODUCTION

At presynaptic compartments of excitatory neurons the cycling in functional activity of SNARE proteins and Rab‐GTPases, along with dynamic regulation by signaling elements, is central to the compartmentalized targeting and Ca^2+^‐regulated exocytotic fusion of synaptic vesicles (SVs) (Binotti, Jahn, Chua, 2016; Rizo, 2018). Rab‐GTPases act as directional regulators on all eukaryotic membrane trafficking. They cycle on/off the donor membrane‐delimited vesicles in a GTP‐dependent manner, recruiting effectors and orchestrating contact with specific target acceptor membranes in a process, that underlies SV membrane targeting, docking and regulation on exocytotic fusion (Binotti et al., 2016; Mignogna & D'Adamo, 2018). Post‐translational geranylgeranylation of the Rab proteins provides the capacity for Rab association with membranes, with the subsequent cycling of Rab proteins onto donor membranes determined by the GTP/GDP status of the Rab protein. Transition of Rab proteins to an active membrane‐associated state is catalyzed by regulatory proteins, such as, guanine‐nucleotide exchange factors (RabGEFs), while GTPase activating proteins (RabGAPs) catalyze the hydrolysis of GTP, returning Rabs to their inactive state (Vetter & Wittinghofer, 2001). In a GDP‐bound state, Rabs may be extracted from membranes by Rab guanine dissociation inhibitor proteins. Evidence indicates Rab‐GTPases function upstream of SNARE proteins in vesicle trafficking and may, in addition to regulating vesicle trafficking, promote membrane fusion by regulation of the activation and engagement of SNARE proteins.

The most abundant Rab proteins in the brain are members of the Rab3‐GTPase family (Rab3A, Rab3B, Rab3C, and Rab3D), which exhibit actions on presynaptic vesicle cycling and vesicular release probability (Fischer von Mollard et al., 1990; Geppert, Goda, Stevens, & Sudhof, 1997; Schluter, Khvotchev, Jahn, & Sudhof, 2002). Enhanced glutamate release with repetitive stimulation and a faster synaptic rundown in Rab3A knockout mice has indicated that Rab3A‐GTP normally serves as a limiting factor to Ca^2+^‐dependent glutamate release. Rab3A was reported essential for normal tetanic induction of long‐term potentiation (LTP) at dentate gyrus/mossy fiber synapses on CA3 neurons (DG/MF‐CA3), which at this synaptic region of the hippocampus presents initially as a form of presynaptic potentiation of synaptic efficacy (Castillo et al., 1997). Moreover a functional linkage between Rab3A and protein kinase A (PKA) in DG/MF‐CA3 LTP was supported based on alteration of PKA’s action on Ca^2+^‐dependent release in Rab3A knockout mice (Lonart, Janz, Johnson, & Sudhof, 1998). In spite of these actions, elimination of all Rab3s in excitatory hippocampal neurons demonstrated only an approximately 30% reduction in mean vesicular release probability, without alteration in the number of primed SV’s (Schluter, Schmitz, Jahn, Rosenmund, & Sudhof, 2004). Rab3B, Rab3C, and Rab3D are closely related to Rab3A and largely, but not completely, act in a redundant fashion in neurons. More recently, using autaptic hippocampal neurons cultured from the quadruple Rab3 isoform knockout, Rab3‐GTPases appear to also be integral to the superpriming of a small subset of the primed, fusion competent SVs at the presynaptic active zone (Schluter, Basu, Sudhof, & Rosenmund, 2006). Superpriming by Rab3‐GTPases may be through binding to the supramolecular complex comprised of Rab3‐interacting molecule 1 (RIM1), RIM‐BP, and Munc13‐1 (Castillo, Schoch, Schmitz, Sudhof, & Malenka, 2002; Schluter et al., 2006; Schoch et al., 2002).

Closely related to the Rab3 gene family are members of the Rab27‐GTPase gene family, which consist of two genes and associated protein isoforms, Rab27A and Rab27B, that differentially localize to dense core secretory granules and synaptic vesicles, respectively (Fukuda, 2003; Pavlos & Jahn, 2011; Yu et al., 2008). Rab27‐GTPases display largely differential expression, with Rab27A expressed in melanocytes, cytotoxic T lymphocytes, various exocrine and endocrine secretory cells and hematopoietic cells but not central neurons (Gomi, Mori, Itohara, & Izumi, 2007; Tolmachova et al., 2004). By comparison, Rab27B primarily localizes to neurons of the mammalian CNS, in addition to platelets and specific gastrointestinal cell types (Gomi et al., 2007; Hendrix et al., 2012; Pavlos & Jahn, 2011). The colocalization of Rab3 and Rab27 on SV’s and secretory granules in several secretory cell types raises questions on functional overlap and perhaps coordinated action. In addition to evidence for co‐localization, evidence has also been provided suggesting Rab3 and Rab27B GTPases may, in part, segregate among SV’s (Pavlos & Jahn, 2011). Within neurons, Rab27B has been reported in complex with Synaptotagmin like protein 1 (Slp1) and CRMP‐2 to participate in anterograde transport of TrkB containing vesicles (Arimura et al., 2009). Yet, mapping specific role(s) of Rab27GTPases within the exocytotic secretory pathway has primarily focused on Rab27A, with results demonstrating overlapping action with Rab3A on vesicle transport, granule docking, and fusion at the plasma membrane in neuroendocrine cells or cell lines (Cazares, Subramani, Saldate, Hoerauf, & Stuenkel, 2014). Rab27 has been reported to reduce SV docking and synaptic release at active zones following anti‐Rab27 antibody injection into the pre‐terminal of the squid giant synapse (Yu et al., 2008). Consistent with partially redundant functional actions of Rab27 and Rab3 GTPases are reports suggesting shared effectors, such as Synaptotagmin‐like proteins (Slp and Rabphilin); Slp‐like proteins lacking C2 homologous regions (Slac and Noc2); vesicle priming proteins and that GTP binding of both Rab27 and Rab3 GTPases involves Rab3GEP (Figueiredo et al., 2008; Fukuda, 2003, 2013). Yet, in vivo affinity measurements have subsequently demonstrated Slp4A, Rabphilin and Noc2 as specific effectors of the Rab27 GTPases relative to Rab3 GTPases (Cheviet, Waselle, & Regazzi, 2004; Fukuda, 2008). In addition, Rab27 and Rab3 GTPases have been reported to exhibit kinetic differences in secretory granule association (Handley, Haynes, & Burgoyne, 2007; Pavlos et al., 2010; Pavlos & Jahn, 2011).

Remarkably, the functional actions of Rab27B in excitatory synaptic transmission and SV exocytosis have remained largely uncharacterized, raising questions on potential compensation on transmission in the Rab3 quadruple knockout. In addition, the role of Rab27B in synaptic facilitation remains unknown. Here to identify the specific actions of Rab27B on synaptic transmission and to establish the extent of functional redundancy or cooperativity with the Rab3‐GTPase family we studied properties of transmission in Rab27B KO mice relative to WT strain controls, as well as on viral rescue of Rab27B in the KO. Specifically, we investigated properties associated with short‐term and long‐term synaptic transmission at both DG/MF‐CA3 and Schaffer collaterals‐CA1 (SC‐CA1) synaptic contacts in hippocampal brain slices. Unexpectedly, the results establish that Rab27B’s role in shaping presynaptic frequency facilitation were directly opposite between the DG/MF‐CA3 and SC‐CA1 synaptic contacts. In addition, we observed Rab27B to be essential for induction of LTP at the DG/MF‐CA3 synapse, but not the SC‐CA1 synapse, even in the continued presence of Rab3‐GTPase expression. Taken together the results are novel in demonstrating that Rab27 and Rab3A are both required for presynaptic LTP, suggesting these closely related Rab‐GTPases exert distinct and perhaps sequentially or parallel activation of downstream effectors.

## 2 MATERIALS AND METHODS

### 2.1 Ethical approval

All animal handling and experimental procedures associated with or performed in this study followed National Institutes of Health (NIH) animal use guidelines and were approved by the Institutional Animal Care & Use Committee (IACUC) at University of Michigan (Approval Number PRO00007460). All investigators understand the ethical principles under which the Journal of Physiology operates and the work complies with the journals animal ethics checklist, Animals were housed in an enriched environment in a 12 hr light–12 hr dark cycle and climate‐controlled room (22°C) with free access to water and food. Mice were deeply anesthetized by isofluorane and then euthanized by decapitation. In addition, all research performed in this study complied with the Institutional Biosafety Committee at University of Michigan (Approval Number IBCA 00000592).

### 2.2 Animals

*Rab27B* *−/−* C57BL/6 mice were initially generated and provided along with strain controls by John A Williams. Briefly, *Rab27B*−/− C57BL/6 mice were bred from Rab27a/b double knockout mice provided by Miguel Seabra and crossed with C57BL/6 from Taconic (Hudson, NY) (Hou, Ernst, Lentz, & Williams, 2016). By subsequent breeding of pups, *Rab27B* −/− and C57BL/6 lines were established and bred as homozygotes. Timely backcrossing of the *Rab27B* −/− mice to C57Bl/6 recipient strain controls, genotyping and generational propagation was performed to sustain consistent breeding success. Genotyping of mice relative to Rab27 was performed by Transnetyx using real‐time PCR (http://www.transnetyx.com). Experiments in this study used mice of either sex, 4‐ to 6‐weeks old, and 15–20 g, unless stated otherwise.

### 2.3 Hippocampal neuronal cultures

Hippocampal neurons from either WT or Rab27B knockout mice were harvested on postnatal day 1(P1) and plated at a density of 60,000 cells on 14‐mm micro well glass‐bottom 35‐mm culture dishes (MatTek Corporation, catalogue no. P35GC‐0‐14‐C). Cultures were maintained for up to 3 weeks in NBActiv4 medium (BrainBits, catalogue no. Nb4‐500) in a tissue culture incubator under controlled conditions (95% O_2_/5% CO_2_, at 37°C). Culture media was replaced every 4 days until the day of the experiment.

### 2.4 Protein fractionation and antibodies

Hippocampi were dissected and homogenized in ice‐cold RIPA buffer (Sigma‐Aldrich, catalogue no. R0278), containing 2× cOmplete^TM^ Protease Inhibitor Cocktail (Sigma‐Aldrich, catalogue no. 11697498001). Lysates were centrifuged at 14,000*g* at 4°C for 10 min. Protein levels of the recovered supernatants from WT and Rab27B mutant mice were then quantified using Bradford assays and protein samples separated by SDS‐PAGE, using 4%–12% Bolt‐Bis‐Tris Plus precast gels (ThermoFisher scientific, catalogue no. NW04120BOX) and NuPage 3%–8% Tris‐Acetate (ThermoFisher scientific, catalogue no. EA03785BOX). After electrophoresis, proteins were wet‐transferred onto nitrocellulose membranes (0.2 µM pore size, GE Healthcare Life Sciences, catalogue no. 10600001) and blocked overnight at 4°C in Odyssey blocking buffer (LI‐COR Biosciences, catalogue no. 927‐40000). Nitrocellulose membranes were incubated for 1 hr at room temperature with the indicated primary antibodies: anti‐Rab27B (1:400, Synaptic Systems Cat# 168 103, RRID:AB_887767), anti‐Rab3A (1:1,000, Synaptic Systems Cat# 107 111, RRID:AB_887770), anti‐Munc13‐1 (1:1,000, Synaptic Systems Cat# 126 103, RRID:AB_887733), anti‐RIM1 (1:1,000, Synaptic Systems Cat# 140 003, RRID:AB_887774), anti‐Tomosyn1 (1:1,000, Synaptic Systems Cat# 183 103, RRID:AB_2619878), anti‐Syntaxin 1A (1:1,000, Synaptic Systems Cat# 110 302, RRID:AB_887846), anti‐Rabphilin 3A (1:1,000, Sigma‐Aldrich Cat# R3026, RRID:AB_1079836), monoclonal anti‐β‐Actin (1:5,000, Sigma‐Aldrich Cat# A2228, RRID:AB_476697), anti‐Munc18‐1 (1:1,000, BD Biosciences Cat# 610,337, RRID:AB_397727), anti‐synaptotagmin1 (1:1,000, Synaptic Systems Cat# 105 105, RRID:AB_1210380), anti‐synapsin1/2 (1:1,000, Synaptic Systems Cat# 106 004, RRID:AB_1106784), anti‐SNAP25 (1:1,000, Synaptic Systems Cat# 111 011, RRID:AB_2619779), anti‐Glutamate Receptor 2 (1:1,000, NeuroMab Cat# 75 002, RRID:AB_2232661). Antibody specificity has been validated by commercial supplier. Secondary antibodies used alone or in combination (1 hr at room temperature) included: IRDye goat anti‐rabbit 680LT (1:15,000, LI‐COR Biosciences Cat# P/N 925‐68021, RRID:AB_2713919), IRDye goat anti‐mouse 800CW (1:15,000, LI‐COR Biosciences Cat# 926–32210, RRID:AB_621842), IRDye goat anti‐guinea pig 680LT (1:15,000, LI‐COR Biosciences Cat# 926‐68030, RRID:AB_10706310). Protein loading and antibody dilutions were determined empirically to ensure linearity in the dynamic range. Western blots were imaged using an Odyssey CLx Infrared Imaging System (LI‐COR model no. 9120) at 84 μm resolution in high‐quality mode and within the linear range of exposure. Fluorescence density was quantified using Image Studio Lite Version 5.2 (LI‐COR).

### 2.5 Immunohistochemistry

WT and Rab27B knockout mice anesthetized with isoflurane were perfused via the left ventricle for 10 min with 4% paraformaldehyde in PBS (0.1 M, pH 7.2). The right atrium was severed to provide drainage. Following excision of the brain and removal of the cerebellum, the brain was immersion fixed in the same fixative overnight. Brains were cryoprotected in PBS with graded 10% to 30% sucrose until they were saturated and then embedded in Shandon M‐1 Embedding Matrix (ThermoFisher Scientific). Coronal sections (15 μm‐thick) containing the hippocampus were cut using a CM1950 cryostat (Leica Microsystems Inc.), thaw‐mounted onto SuperFrost Plus glass slides (ThermoFisher Scientific) and stored at −80°C until use. For fluorescence immunohistochemistry, tissue sections were blocked in 5% normal goat serum/0.3% Triton‐X100/PBS for 1 hr at room temperature, followed by incubation overnight at 4°C in 2% normal goat serum/0.3% Triton‐X100/PBS containing rabbit polyclonal anti‐Rab27B antibody (1:1,000, Synaptic Systems Cat# 168 103, RRID:AB_887767), and 1:250 monoclonal anti‐synapsin 1 conjugated to Oyster 488 (Synaptic Systems Cat# 106 011C2, RRID:AB_10805139). After PBS rinses, sections were incubated in secondary antibody 1:500 Alexa Fluor 594‐conjugated goat anti‐rabbit IgG (Thermo Fisher Scientific Cat# A‐11012, RRID:AB_2534079), in the same solution as primary antibodies for 1 hr at room temperature. Sections were rinsed in PBS and mounted with glass coverslips and Prolong Gold with DAPI (ThermoFisher Scientific). For panorama confocal immunohistochemistry, images of whole hippocampi were taken on a Nikon A1 confocal microscope with a 20× dry (0.75 NA) objective and Nikon Elements software (version 4.5.1). Fluorescence intensity was adjusted to near saturation of Rab27B and synapsin signals in WT dentate gyrus and CA3 regions. Fluorescence signals were acquired sequentially with the following filter sets: DAPI, blue 425–475 nm, synapsin‐Oyster, green 500–530 nm, and Rab27B‐AlexaFluor594, red 553–618 nm. The scan parameters were set to 1024 × 1024 pixel resolution, 6 airy units for confocal aperture (9.45 µm optical sectioning), zoom 3×, 2 line averaging, and 0.5 frames per second. Panoramic views through the hippocampi were captured by large format scans in XYZ arrays that had 5% overlap in XY and z‐step sizes of 1.25 µm across 3 steps. Final photomicrographs were maximum projection images collapsed over the 3 z‐steps. Rab27B expression and synapsin staining in the CA1 region was also photographed at higher magnification (60× water immersion lens) using a Fluoview 500 Olympus confocal microscope at near saturation of the signals in this region. Rab3A expression was also examined in the CA1 and CA3 regions. In this case, 300 μm‐thick Vibratome sections of the hippocampus region, immersion fixed in 2% paraformaldehyde in PBS for 1 hr, were cryoprotected and frozen. Cryosections (8–10 μm‐thick) were prepared with the CM1950 cryostat and immunostained using sequential incubations of 1:250 monoclonal anti‐Rab3A antibody (Synaptic Systems Cat# 107 111, RRID:AB_887770), PBS rinses, 1:500 secondary antibody Alexa Fluor 594‐conjugated goat anti‐mouse IgG and, following rinsing in PBS, 1:250 synapsin‐Oyster 488.

### 2.6 Electron microscopy

The fixation protocol followed an established procedure (Schikorski & Stevens, 1997). Briefly, anesthetized WT and Rab27B knockout mice were perfused with oxygenated saline followed by 10 min perfusion with 4% glutaraldehyde in 100 mM phosphate buffer, pH 7.2. The brain was removed and fixed overnight in the same fixative. After rinsing, 300 µm‐thick Vibratome slices of the brain, cut through the hippocampus region, were post‐fixed for 1 hr at 4°C in a mixture of 1% OsO_4_ and 1.5% K^+^‐ferrocyanide. The slices were rinsed, bisected to make half brains, dehydrated and flat embedded in Epon. Ultrathin sections of the CA1 region where examined after staining with lead citrate and uranyl acetate with a JEOL JSM 1400 electron microscope. Images of synapses were recorded digitally at a print magnification of 68,900× and processed in Adobe Photoshop CS6 (Adobe Photoshop, RRID:SCR_014199). Synaptic vesicle diameters were determined using the Photoshop measurement software. Synaptic vesicles were analyzed in synapses of 2 WT and 2 Rab27B knockout mice. For CA1 a total of 17 WT and 18 KO synapses were analyzed and diameters of 113 WT and 130 Rab27B KO synaptic vesicles were measured. In CA3, 221 WT and 234 Rab27 KO synaptic vesicles were measured from 22 and 17 synapses, respectively.

### 2.7 Acute hippocampal slices

On the day of the experiment mice were anaesthetized with isoflurane and euthanized by decapitation. The brain was removed quickly and transferred onto a Petri dish filled with ice‐cold cutting solution (in mM: 2.5 KCl, 1 CaCl_2_, 4 MgSO_4_, 1.6 NaH_2_PO_4_, 4 MgCl_2_, 10 glucose, 215 sucrose, 26 NaHCO_3_, pH 7.4), where the hippocampi were carefully dissected from the brain. Hippocampi were then embedded into 4% low melting point agar and 300‐µm thick transversal hippocampal slices were prepared using a VT1000 Vibratome (Leica). Slices were collected and transferred into a slice interface chamber filled with room temperature extracellular artificial cerebrospinal fluid (ACSF), in mM: 124 NaCl, 2.5 KCl, 1.3 MgSO_4_, 2.5 CaCl_2_, 1 NaH2PO_4_, 10 glucose, 26 NaHCO_3_, pH 7.4, for a minimum of 1 hr prior to beginning electrophysiological recordings. Both cutting solution and ACSF were continuously equilibrated with 95% O_2_ & 5% CO_2_ gas mixture.

### 2.8 Brain slice electrophysiology

Field excitatory synaptic potentials (fEPSPs) were recorded in acute hippocampal slices placed in a perfused slice interface chamber using glass pipettes (1–2 MOhm) placed along the cell layer appropriate to the synaptic hippocampal region studied. For stimulation, monophasic current pulses, 0.1–0.5 ms duration, were delivered through a bipolar tungsten electrode (World Precision Instruments, model TST33CO5KT) via a constant current stimulus isolation unit (World Precision Instruments, model A360). Stimulus intensity was gradually adjusted to yield post‐synaptic responses between 0.5–1 mV for CA1 and responses between 0.1 mV and 0.5 mV for CA3. Electrophysiological signals were recorded using a HEKA patch clamp EPC10 USB amplifier and digitized at sample rate of 10 kHz, with subsequent filtering at 2 kHz via a low‐pass filter. Extracellular ACSF was pre‐heated to 25 ± 1°C with an in‐line temperature control (Harvard Apparatus, model TC‐324C), and perfused by gravity at a rate of ~2 ml per minute.

### 2.9 Whole‐cell recordings

Miniature excitatory synaptic currents (mEPSC) were recorded from pyramidal hippocampal neurons in culture (20–24 days in vitro), using glass electrodes (~4 MOhm) filled with, in mM: 105 Cs‐gluconate, 5 MgCl_2_, 0.2 EGTA, 40 HEPES, 2 ATP, 0.3 GTP; and with ACSF as bath solution. For these experiments, ACSF was supplemented with 1 µM TTX and 10 µM Bicuculine. Recordings were only included in analysis if holding current was maintained under 250 pA, and for cells with membrane potential more polarized than −55 mV and series resistance ≤25 MOhm. Electrophysiological recordings were sampled at 10 kHz and filtered at 2.8 kHz. A value of 5 pA was defined as threshold for event detection using TaroTools software.

### 2.10 Lentiviral particle generation

To generate custom Lentiviral particles, the open reading frame (ORF) of the complementary DNA (cDNA) encoding the Human Rab27B gene (OriGene, catalogue no. RC206519) was subcloned into the pLenti‐N‐tRFP Tagged Cloning Vector (OriGene, Catalogue no. PS100077). Plasmids were amplified using One Shot™ Stbl3™ chemically competent *Escherichia coli* (Invitrogen, catalogue no. C737303), and purified with QIAGEN endotoxin‐free plasmid Maxi Kit (QIAGEN, catalogue no. 12362). The pLenti‐EV‐GFP‐VSVG plasmid was purchased as a ready‐made stock. All Lentiviral particles used for this study were generated at the vector core facility of the University of Michigan (https://brcf.medicine.umich.edu/cores/vector/).

### 2.11 Stereotaxic injections

Mice were prepared following the guidelines on the performance of survival surgery in rodents of the Animal Care & Use Program of the University of Michigan and with IACUC (University of Michigan) protocol approval. Anesthesia was induced with isoflurane at 3%–3.5% saturation (500 ml/min) and maintained at 1.5%–2.5% (~80 ml/min) using a low‐flow anesthesia system (Kent Scientific Corporation, SomnoSuite). Carprofen was administered subcutaneously at a concentration of 2.2 mg/kg before starting the surgical procedure. In the stereotaxic apparatus (Leica, Angle Two), an incision along the midline of the head was made with surgical scissors, and the skull was exposed. Then, a craniotomy was performed on the skull above the injection site(s) defined by the following coordinates: ±1.4 mm ML, −2.3 mm AP, and −1.9 DV, relative to bregma and lambda. Bilateral or unilateral stereotaxic injections were performed, with a total volume of 2 μl of virus (~1 × 10^7^ transduced units/ml) injected per side at a rate of 0.2 μl/min. The needle remained in the injection site for an additional 2 min after the injection and was removed with extreme care afterwards to avoid spillover. At the end of the procedure, the incision was sutured with sterile plain gut sutures (Ethicon, catalogue no. 774G), and mice were monitored until they become ambulatory. A post‐operative surgical record was kept for each mouse for 10 days after surgery. Mice were euthanized on the 5th week post‐surgery either for imaging or electrophysiology experiments.

### 2.12 Data analysis and statistics

Two‐tailed, paired or unpaired Student's *t*‐test were used to assess statistical significance between groups for comparisons of samples with equal variance (*α* = .05), whereas two‐way analysis of variance (ANOVA) was used for multiple comparisons. Data quantification and analysis were performed using IGOR Pro (Wavemetrics), Axograph (by John Clements), GraphPad prism (version 7) and Microsoft Excel (Microsoft). Data summaries in text and all figures presented as means ± standard deviation (*SD*) and include statement of relevant *n* values.

## 3 RESULTS

### 3.1 Rab27B Expression in the mouse hippocampus

**FIGURE 1:**
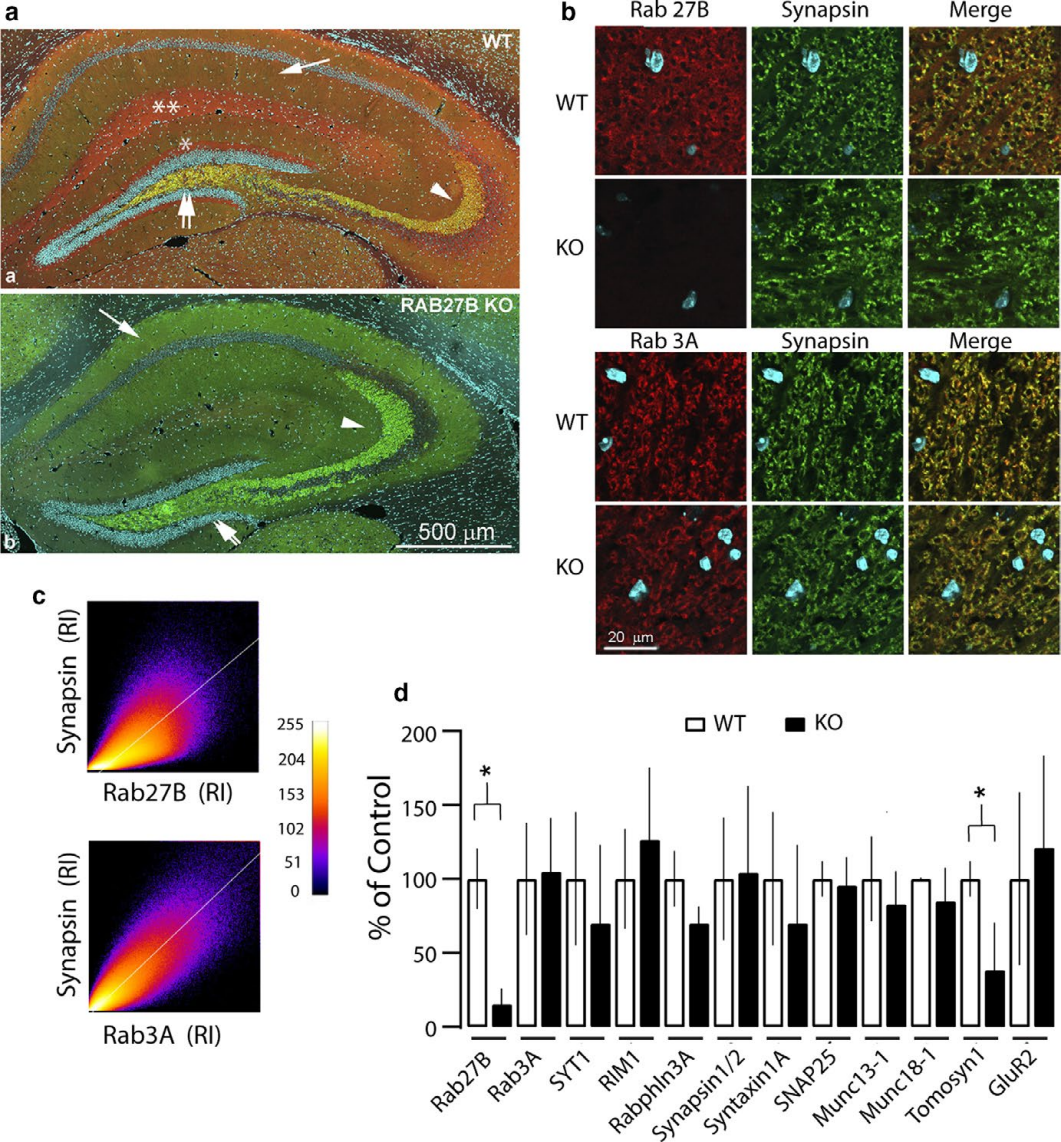
Rab27B expression and synaptic localization in hippocampal regions. (A) Overlay of confocal panoramic images of Rab27B (red) and synapsin (green) immunostaining in (a) wild type and (b) Rab27B knockout (KO) mouse hippocampus cryostat sections. Rab27B staining intensity in wild type CA3 (arrowhead) CA1 (arrow) and dentate gyrus (double arrows) regions and colocalization (yellow) with synapsin. Rab27B staining intensity, but not synapsin staining, apparent in lateral performant path (*) and stratum lacunosum‐moleculaire (**) regions (*n*, WT = 3, KO = 3). Rab27B staining is absent in the knockout condition. Nuclei are stained with DAPI. (B) Rab27B (red) and synapsin (green) immunostaining in the CA3 region of wild type and Rab27B KO hippocampus (upper panel). Rab27B staining in the wild type co‐localizes (yellow) with synapsin, whereas it is absent in the KO. Rab3A staining (lower panel) also co‐localizes with synapsin in wild type, with staining intensity only slightly reduced in the Rab27B KO. (C) Pixel by pixel relative immunofluorescence intensity (RI) relationships of synapsin to Rab27B (upper) and synapsin to Rab3A (lower) scaled according to Intensity gradient map (right). Pearson *R* correlations: .85, Rab3A and synapsin; .69, Rab27 and synapsin. (D) Relative expression levels defined by immunoblot analysis of presynaptic proteins in wild type and Rab27B knockout mice. Signal intensities were normalized to actin and expressed as percentage of control. In addition to Rab27B, Tomosyn 1 was the only other synaptic protein demonstrating a significant reduction in expression (*p = .0241) in the Rab27B KO samples (*n*, >5 per protein)

While Rab27B‐GTPase has been localized to synaptic vesicle membranes at presynaptic terminals (Pavlos & Jahn, 2011), as well as to pituitary, pancreas, and gastric acid‐secreting parietal cells (Hendrix et al., 2012), its spatial distribution in the intact hippocampus and comparison to Rab3A expression has not been fully established. To define regional expression within brain slices of the mouse hippocampus we performed immunohistochemistry and recorded staining in low magnification confocal panoramas. Figure 1Aa compares immunofluorescence signals in the WT hippocampus associated with Rab27B (red) and the presynaptic marker synapsin (green). To enhance anatomical distinction within the slice nuclei of the cell bodies were stained with DAPI (blue). Rab27B demonstrated widespread distribution across the hippocampus, with strong staining intensity and colocalization with synapsin notable within the stratum radiatum synaptic region of CA3. Colocalization was also observed within the statum radiatum and stratum oriens regions of CA1, although of lower intensity for Rab27B. Note that the lateral perforant path of layer II entorhinal projections and the stratum lacunosum‐moleculaire CA1 region, which receives inputs from layer III of the entorhinal cortex, demonstrate strong Rab27B immunoreactivity, but were largely unreactive for synapsin. By comparison, immunoreactive staining for Rab27B was, as expected, completely absent in the Rab27B KO, while strong immunolabeling of synapsin was maintained (Figure 1Ab). Immunohistochemical co‐localization of Rab27B and Rab3A with synapsin was well delineated at higher magnification within the CA3 region of WT mice (Figure 1B). Indeed, pixel by pixel analytical analysis demonstrated a Pearson R correlation of .69 for Rab27B and synapsin (Figure 1C). Moreover immunoreactive colocalization of Rab3A and synapsin persisted in Rab27B knockout mice (Pearson *R* correlation = .85), although the immunoreactive intensity for Rab3A was slightly reduced relative to that in the WT control (Figure 1B).

To further establish if Rab27B KO mice exhibited significant alterations in hippocampal synaptic protein expression relative to WT mice analysis was performed on hippocampal lysates by quantitative SDS‐PAGE and western blotting for a subset of synaptic proteins, including selected synaptic effectors of Rab‐GTPases (Figure 1D). The results demonstrate a near complete elimination of Rab27B immunoreactivity in the Rab27B KO relative to WT control. Notably, Rab3A expression in the Rab27B KO was unaltered from WT control. In addition, comparison of WT and Rab27 KO lysates demonstrated no significant differences in synaptic protein expression levels, except for the V‐SNARE domain containing protein Tomosyn‐1. Tomosyn‐1, which is reported to act as a negative regulator on presynaptic release (Hatsuzawa, Lang, Fasshauer, Bruns, & Jahn, 2003; Yizhar et al., 2004), be subject to CDK5 phosphorylation and interact with Rab GTPases (Cazares et al., 2016), was reduced by approximately 62% (WT, 0.6259 ± 0.07; KO = 0.2389 ± 0.18).

### 3.2 Comparison of synaptic morphology and spontaneous neurotransmitter release at hippocampal synapses in WT and Rab27B knockout mice

**FIGURE 2:**
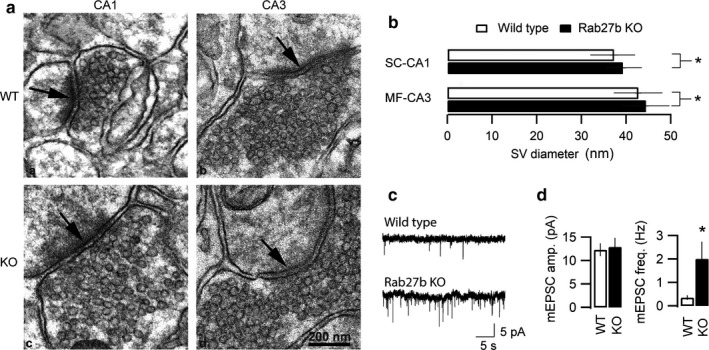
Comparison of synaptic vesicle morphology and spontaneous neurotransmitter release of control and Rab27B KO neurons. (A) Representative transmission electron micrographs of SC‐CA1 and MF‐CA3 hippocampal synapses (arrow) of wild type (upper) and Rab27Bb knockout (lower) mice (*n*, WT = 3, KO = 3). (B) Comparison of average synaptic vesicle diameter in SC‐CA1 (upper) and in MF‐CA3 (lower) presynaptic terminals in WT and Rab27B KO. Synaptic vesicles are slightly but significantly larger in both SC‐CA1 (*p* = .013) and MF (*p* = .001) terminals of Rab27B KO mice compared to control. (C) Representative records of spontaneous mEPSCs in hippocampal neurons cultured from wild type and Rab27B KO mice. (D) Averaged mEPSC amplitude and frequency of recordings from WT and KO neuronal cultures. Statistical analysis revealed a significant fourfold increase in the mEPSC frequency in Rab27B KO compared to control without a significant change in the mEPSC amplitude (*n*, WT = 9, KO = 10, over three separate neuronal cell preparations)

While Rab GTPases serve a central function in trafficking of membrane delimited vesicles in eukaryotic cells, they may in certain cases guide vesicle biogenesis and maturation (Barr, 2013; Schonn et al., 2010). Indeed, the Rab27B KO mouse was reported to have reduced diameter of zymogen granules in pancreatic acinar cells (Hou et al., 2016). To examine potential actions of Rab27B on synaptic morphology and SV diameter we performed high‐resolution electron microscopy (EM) analysis on hippocampal synapses of WT and Rab27B knockout mice. Figure 2A shows representative EM micrographs of DG/MF‐CA3 and SC‐CA1 hippocampal synapses in WT and Rab27B KO mice. Average diameters of SV in SC‐CA1 synapses were 37.2 ± 0.92 nm in WT mice versus 39.2 ± 0.71 nm in Rab27B knockout mice. At MF‐CA3 synapses the average diameters were 42.7 ± 0.71 nm in WT mice versus 44.4 ± 0.65 nm in Rab27B knockout mice. The differences in averaged SV diameter between WT and Rab27B KO were significant with larger SVs in Rab27B knockout compared to WT mice (*p = .013 and **p = .001, respectively; two‐tailed Student's *t*‐test, homoscedastic) (Figure 2B).

In neurons, changes in synaptic vesicle diameter may translate to differences in neurotransmitter vesicular content and hence alterations in the amplitude and duration of quantal miniature postsynaptic potentials. The observed differences in SV diameter with Rab27B KO at the SC‐CA1 and MF‐CA3 synapses indicate respectively, approximate 11%–15% differences in SV volume. To determine if Rab27B KO resulted in functional differences in individual quantal release properties, whole‐cell voltage clamp electrophysiological measurements of spontaneous miniature post‐synaptic current responses (mEPSC) were performed on cultured hippocampal neurons from WT and Rab27B KO mice (Figure 2C). As shown in the representative electrophysiological recordings and in averaged data, no significant difference in mEPSC amplitude was found between neurons cultured from WT or Rab27B KO hippocampi (WT, 12.3 ± 0.66 pA; Rab27B KO, 12.93 ± 0.92 pA), indicating that the observed changes in SV volume were not translated into alterations in SV neurotransmitter content. Notably, however, Rab27B KO resulted in a significant and substantial (>5‐fold) increase in the frequency of spontaneous mEPSC, with events increasing from 0.35 ± 0.05 Hz in WT to 2.01 ± 0.36 Hz in Rab27B KO cultures. These data suggest that Rab27B may be involved in regulating Ca^2+^‐independent transitions between priming/docking steps, which take place prior to SV fusion and neurotransmitter release.

### 3.3 Evoked neurotransmitter release in acute hippocampal slices of WT and Rab27B knockout mice

**FIGURE 3:**
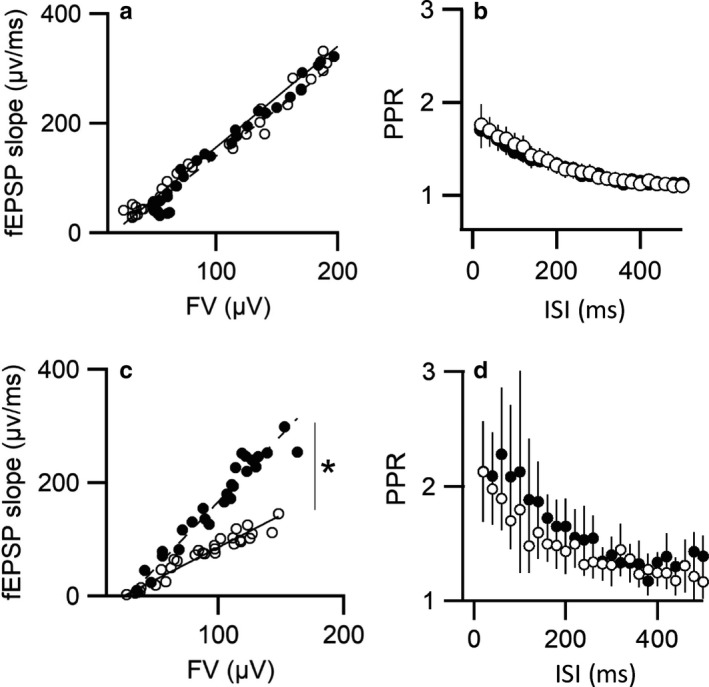
Comparison of properties of evoked synaptic transmission in hippocampal slices. (a) Input‐output relationships in SC‐CA1 synapses of wild type (○) and Rab27B KO (●) mice. Synaptic responses are not significantly different between wild type and Rab27B KO mice (animal *n*, WT = 8, KO = 7, *p* = .35). (b) Comparison of paired‐pulse ratio (PPR, R2/R1) as a function of inter‐stimulus interval in SC‐CA1 synapses. The average PPR decay time constant was estimated by exponential fit of data. Statistical analysis revealed no differences in kinetics (animal *n*, WT = 12, KO = 17, *p* = .35). (c) Input‐output relationships in MF‐CA3 synapses of wild type (○) and Rab27B KO (●) mice. Rab27B KO demonstrated a statistically significant increase in input‐output relationship with respect to WT controls (animal *n*, WT = 8, KO = 7, *p = .03). (d) Analysis of paired‐pulse ratio in MF‐CA3 synapses. No statistically significant differences were found in the average calculated PPR decay time constants between WT and Rab27B KO mice, (animal *n*, WT = 13, KO = 8, *p* = .4)

To address whether Rab27B exerts a principal regulatory role within evoked neurotransmitter release, we next examined properties of synaptic transmission at SC‐CA1 and DG/MF‐CA3 synapses in acute hippocampal slices from WT and Rab27B KO mice. To define input–output relations, synaptic responses were elicited by graded electrical stimulation appropriate for each synaptic region and the slope of the field excitatory postsynaptic potential (fEPSP), which is proportional to the amount of neurotransmitter released, was plotted as a function of the presynaptic fiber volley amplitude. For SC‐CA1 synapses the average slope was 1843 µV/ms in WT and 1715.4 µV/ms in Rab27B knockout mice (Figure 3a). Statistical analysis revealed that SC‐CA1 field synaptic responses are not significantly different between WT and Rab27B knockout mice (*p* = .35). By comparison, at the MF‐CA3 synaptic connection, the average slope was 1,142.5 µV/ms in WT and 2,336.3 µV/ms in KO, revealing a significant (*p* = .03) doubling in the average slope in Rab27B with respect to WT controls (Figure 3c).

To provide an indirect estimator of the effect of Rab27B KO relative to WT on neurotransmitter release probability, we next measured paired‐pulse ratio (PPR) responses at both SC‐CA1 and DG/MF‐CA3 synaptic sites (Zucker & Regehr, 2002). PPR was performed by delivering two consecutive electric shocks separated by an inter‐stimulus interval (∆t) over a range from 20 to 500 msec. The PPR was computed using the fEPSP amplitude of the second pulse (R_2_) divided by the fEPSP amplitude of the first (R_1_). The data was then analyzed by fitting an inverted exponential function to the resulting PPR relationships and by comparing the maximum facilitation and the decay time constant. As shown in Figure 3b there was little observable difference between the PPR ratio data for WT and Rab27 KO at SC‐CA1 synapses across the range of inter‐stimulus intervals. Both maximum PPR values (WT = 1.840.29 ± 0.29 and KO = 1.8 ± 0.23) and averaged time constants for PPR decay (WT, 166.4 ± 7.76 ms; Rab27B KO 164.36 ± 9.72 ms) were not significantly different. For MF‐CA3 synapses, maximum PPR values were WT = 2.125 ± 0.30 and KO = 2.131 ± 0.44; and the averaged PPR time constants of decay (131.79 ± 19.3 ms for WT and 204.4 ± 50.6 ms for KO) were also not significantly different. Thus, PPR was nearly identical between WT and Rab27B knockout mice at both synaptic regions, suggesting that deletion of Rab27B it is not likely to affect initial evoked release probability in hippocampal synapses. However, a difference in input‐output relations between WT and KO at the MF‐CA3 synapse not being reflected in a measurable difference in the PPR may result from the fEPSP’s for WT and KO at the half‐max stimulation intensity used for PPR being similar.

### 3.4 Effect of Rab27B knockout on frequency facilitation in hippocampal synapses

**FIGURE 4:**
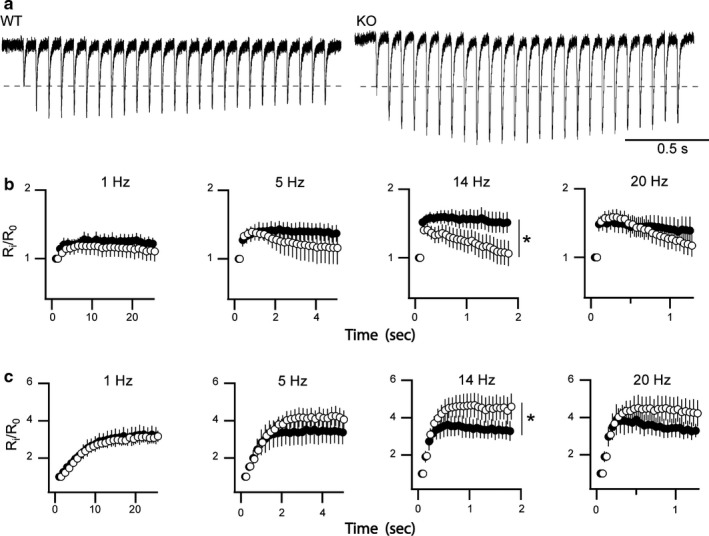
Effects of Rab27B KO on frequency facilitation at SC‐CA1 and MF‐CA3 synapses. (a) Representative recordings comparing kinetics of evoked EPSP facilitation of SC‐CA1 to a train of 25 stimuli applied at 14 Hz in WT and Rab27B KO hippocampal brain slices. (b) Comparison of facilitation responses in SC‐CA1 synaptic region across a range of applied frequencies in both WT (○) and Rab27B KO (●) brain slices. A significant increase in facilitation was observed in the Rab27B KO relative to WT at 14 Hz (animal *n* = 12, *p = .007) with a similar trend appearing at 1, 5 and 20 Hz, although statistical significance was not present (animal *n*, WT = 12, KO = 12, *p* = .31, *p* = .28, and *p* = .7, respectively). (c) Frequency facilitation at MF‐CA3 synapses trended toward reduced facilitation as stimulation frequency increased in Rab27B KO slices compared to WT. Statistically the frequency response for 1, 5, and 20 Hz was not significantly different between conditions (*p* = .74, *p* = .18, and *p* = .09, respectively), while attenuation of facilitation was significantly different between Rab27B KO and WT at 14 Hz (animal *n*, WT = 13, KO = 9, *p = .0243)

Rab3A has been reported central to a step in SV priming based, in part, on observed differences in frequency facilitation (FF) to repetitive stimulation for WT and Rab3A KO deficient mice at the SC‐CA1 synaptic region in acute hippocampal slices (Geppert et al., 1994). In WT mice, repetitive stimulation of the SC pathway at 14 Hz produced frequency facilitation (FF) of the fEPSC amplitude in CA1 pyramidal neurons, whereas in Rab3A knockout mice the same treatment produced FF followed by synaptic depression. To establish if this FF effect is unique to the neuronal Rab3A GTPase, or if Rab27B may exhibit a redundant function we tested if Rab27B elimination exerted similar actions on FF by applying trains of 25 stimuli at different frequencies (e.g., 5, 10, 14, and 20 Hz). Figure 4a shows representative fEPSP responses to 14 Hz FF stimulation recorded from SC‐CA1 synaptic region of WT and Rab27B KO acute hippocampal slices. Repetitive stimulation of the SC of WT mice generated a biphasic response, an initial increase in the amplitude of the fEPSP followed by a progressive decay, which may result from a frequency‐related deficit in SV replenishment. In the Rab27B knockout mice robust facilitation was found to persist throughout the stimulus train and with no apparent decay. Figure 4b shows averaged data collated over a range of stimulation frequencies. The average normalized response at the end of the stimulus train for 14 Hz stimulation were WT = 1.12 ± 0.19 versus KO = 1.49 ± 0.14. These data indicate that Rab27B may normally act as a temporal negative regulator on the neuronal SV exocytotic process. That is, the Rab27B KO displays a ~30% increase in the relative amplitude of fEPSPs compared to control values. It is also important to note that although differential action on FF observed at 14 Hz between WT and Rab27B KO was statistically significant only at 14 HZ, a similar trend was observed across the applied frequencies.

Remarkably, examination of this FF pathway at the DG/MF‐CA3 synaptic pathway resulted in a directly opposite facilitation phenotype to that observed in the SC‐CA1 pathway. As shown in Figure 4c, Rab27B‐deficient mice showed significantly reduced frequency facilitation relative to WT controls in response to repetitive 14 Hz stimulation of MF fibers. A similar trend was observed across the applied frequencies, except for 1 Hz. Note that repetitive stimulation of MF did not cause synaptic depression in WT at the frequencies tested and only slight depression in the Rab27B KO slices at frequencies of 14 Hz and 20 Hz. Experiments were performed without block of GABA receptors for both conditions suggesting the observed differences are unlikely to be accounted for by recruitment of inhibitory synapses, unless recruitment would be more pronounced in the Rab27B KO condition. The opposing differences in frequency facilitation observed with Rab27B KO relative to control at the SC‐CA1 and DG/MF‐CA3 synaptic regions demonstrate that actions of Rab27B, as shown previously for Rab3A, are specific to the synapse type, even among the excitatory synaptic connections within the hippocampus.

### 3.5 Effect of Rab27B knockout on NMDA‐ and PKA‐dependent forms of long‐term potentiation

**FIGURE 5:**
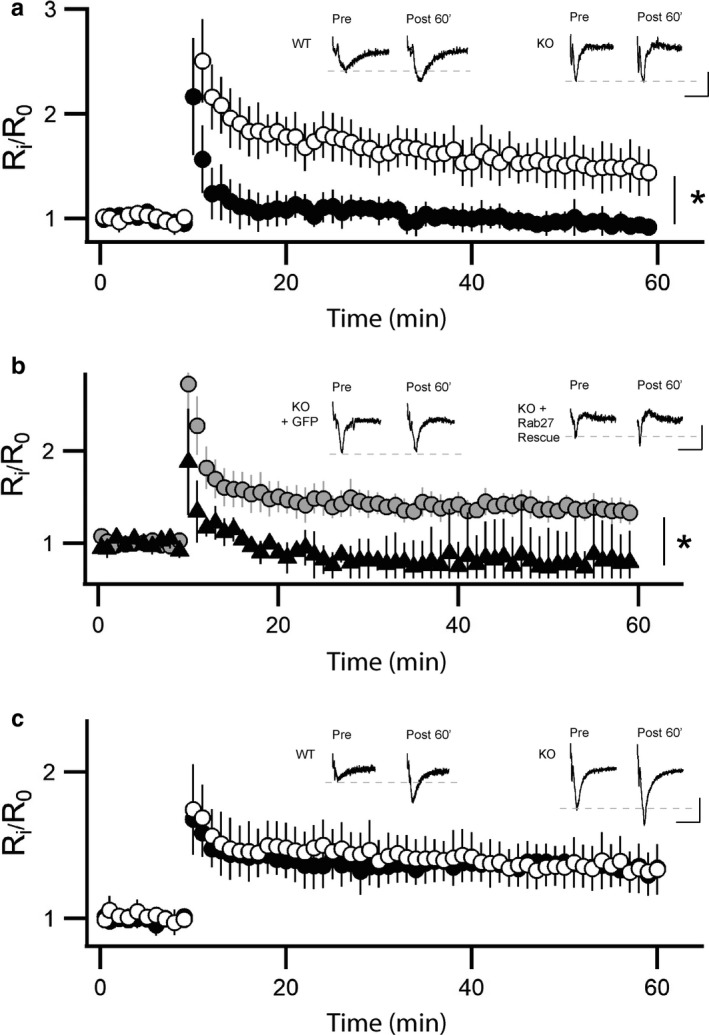
Effects of Rab27B KO on long‐term potentiation at SC‐CA1 and MF‐CA3 synaptic regions. (a) Rab27B KO eliminates capacity to induce LTP at MF‐CA3 synapses (animal *n* WT = 6, KO = 5, *p = .0126). Averaged LTP induction and time course is shown for wild type (○) and Rab27B knockout mice (●). (b) Lentiviral microinjection and overexpression of human Rab27B in the dentate gyrus of Rab27B KO mice (●) rescued LTP at MF‐CA3 synapses, whereas overexpression of GFP (▲) failed to rescue LTP (animal *n*, KO + Rab27B=6, KO + GFP=3 *p = .0015). (c) LTP induction and time course are undistinguishable between Rab27B KO (●) and wild type controls (○) at SC‐CA1 synapses (animal *n*: WT = 10, KO = 11). (a–c) Representative data traces are recordings averaged over three consecutive data points per condition from single hippocampal slices

The hippocampus is known for its ability to express long‐lasting forms of synaptic plasticity, such as long‐term potentiation (LTP) (Lomo, 2018). It has been reported that Rab3A knockout selectively eliminates presynaptic PKA‐dependent long‐term potentiation in MF synapses (Castillo et al., 1997; Lonart et al., 1998), but does not affect postsynaptic NMDA‐dependent LTP in CA1 (Geppert et al., 1994). We next tested if Rab27B exerts similar or distinct actions on the induction and properties of LTP at the DG/MF‐CA3 synaptic region. For these investigations LTP was induced in WT and Rab27B KO slices by stimulating MF with two trains of 5 s duration at 25 Hz separated by an inter‐train interval of 20 s. Importantly, the results demonstrated that MF stimulation completely failed to induce presynaptic MF‐CA3 LTP in slices from Rab27B knockout mice, while LTP was reliably induced in hippocampal slices of WT mice (Figure 5a). Notably, as the Rab27B KO retains expression of Rab3A GTPase these results demonstrate that Rab3A and Rab27B are both essential and fail to functionally compensate within LTP induction. Importantly, we next evaluated if lentiviral driven expression of Rab27B in the Rab27B KO would be able to rescue induction of LTP and indicate apparent specificity of action Rab27B. For these experiments, lentivirus encoding human Rab27B (hRab27B) was stereotaxic microinjected into the right or left dentate gyrus, with a lentiviral control virus encoding GFP independently injected into the contralateral dentate gyrus of the same mouse. As shown in Figure 5b, paired recordings of the hippocampal slices resulted in a near complete rescue of LTP induction and maintenance with overexpression of the human Rab27B, whereas overexpression of GFP failed to rescue LTP induction in the Rab27B KO background.

To assess if Rab27B may also exert a role in NMDA‐dependent LTP in CA1, we induced LTP in WT and Rab27B KO slices by stimulation of the SC pathway with 4 trains of 1 s duration at 100 Hz separated by an inter‐train interval of 20 s. The results demonstrated that the stimulus reliably elicited robust LTP in both WT and Rab27B knockout mice. Moreover quantification and comparison of the magnitude and kinetics of the resulting LTP revealed no significant differences between WT and Rab27B mutant mice (Figure 5c). These findings functionally replicate a prior report demonstrating sustained electrical activity induced LTP at the CA1 region in Rab3A‐GTPase KO mouse model (Geppert et al., 1994). In summary, while Rab27B and Rab3A GTPases are both independently required for LTP induction in the DG/MF‐CA3 pathway neither GTPase appears to be essential for activity induced LTP within the CA1 synaptic region.

## 4 DISCUSSION

Rab‐GTPases are central regulators of membrane trafficking and directly involved in the regulation of exocytotic pathways in neurons, endocrine, and in neuroendocrine cells (Barr, 2013; Binotti et al., 2016). Within the presynaptic compartment the role of Rab3 GTPases has been extensively investigated, yet in spite of considerable attention many temporal and spatial details of their actions and identification of relevant protein complexes that mediate different functional actions remain only partially defined. Rab27B‐GTPase, a close evolutionary relative of Rab3 (41 to 44% sequence homology for human Rab3’s) that overlaps in reported effectors with Rab3 and which is presynaptically localized on synaptic vesicles, remains undefined functionally in the synaptic exocytotic process. The findings of the present study are novel in demonstrating that Rab27B exerts a regulatory role on synaptic efficacy as illustrated by actions on frequency facilitation and, remarkably, with observed actions being opposite at the DG/MF‐CA3 than at the SC‐CA1 hippocampal synaptic regions. These findings demonstrate that Rab27B actions are synapse specific within the hippocampus. Notably, deficiency of Rab27B resulted in a complete elimination of LTP at the DG/MF‐CA3 synaptic region, in spite of the continued presence of Rab3A, while exhibiting no observable action on LTP at the SC‐CA1 synapse. Importantly, overexpression of human Rab27B via lentiviral microinjection in the DG of Rab27B knockout mice rescued LTP at the DG/MF‐CA3, demonstrating specificity of the effect to Rab27B. The loss of LTP at the DG/MF‐CA3 synapse with independent deletion of either Rab3A, as previously reported, or Rab27B, as shown here, indicates both Rab‐GTPases are essential to LTP. Our data, thereby, raise a novel perspective of dual and non‐overlapping roles of these Rab‐GTPases in the exocytotic process underlying LTP at the DG/MF‐CA3 synapse. While it is known that several Rab‐GTPases may localize to the same distinct subcompartments, and operate in different spatio‐temporal microdomains (Sonnichsen, Renzis, Nielsen, Rietdorf, & Zerial, 2000), direct evidence for Rab3 and Rab27 GTPases acting in non‐complementary but essential successive or distinct steps in induction of LTP has not been previously revealed. Notably, independent and successive actions of these GTPases has been reported for exocytosis of the acrosome of the mature spermatozoon where active Rab27 increases the exchange of GDP for GTP on Rab3A, likely by Rab27 recruiting a Rab3GEF (Bustos, Lucchesi, Ruete, Mayorga, & Tomes, 2012). Moreover in support of this model a functional relationship between the Rab3 exchange factor homolog AEX‐3 and Rab27 has been reported in C. elegans (Mahoney et al., 2006).

Both Rab3A and Rab27A have been reported to associate with secretory granules (SGs) and regulate Ca^2+^‐dependent secretion in neuroendocrine cells, although the high structural homology and potential promiscuity of effectors between these Rab families has created difficulty assigning specific functional roles (Cazares et al., 2014; Fukuda, 2008; Pavlos & Jahn, 2011; Binotti et al., 2016). Within neuroendocrine cells Rab27A and Rab3A promote vesicle docking and stimulated secretion, as individual silencing of each Rab GTPase reduced the number of docked vesicles (Tsuboi & Fukuda, 2006). In addition, the effects were further accentuated when both GTPases were simultaneously silenced, suggesting cooperative action. In pancreatic beta cells, Rab27A is essential to functional generation of an immediate releasable pool of secretory granules, while Rab3 GTPases functioned in the kinetically rapid refilling of the readily releasable pool (Cazares et al., 2014). On the other hand, Rab3A has also been reported to negatively regulate secretion in neurons (Fischer von Mollard et al., 1990; Johannes et al., 1994, 1998), pancreatic beta cells (Regazzi et al., 1996), and adrenal chromaffin cells (Holz, Brondyk, Senter, Kuizon, & Macara, 1994). While both Rab27 and Rab3 GTPases are coupled to exocytosis, they are kinetically distinct in cycling between the cytosol and vesicle membranes (Handley et al., 2007; Pavlos et al., 2010). Rab3A cycles continuously with Rab3A‐GTP hydrolysis associated with the timing of regulated exocytosis in neurons, but this link is perhaps not as tight for secretory granules (Handley et al., 2007). By comparison, Rab27 localizes to secretory granules with little, or kinetically slow, exchange with the cytosol (Handley et al., 2007; Pavlos et al., 2010).

Our data reveal that Rab27B, as well as Rab3A, are widely expressed across the murine hippocampus and that both GTPases colocalize with synapsin, a presynaptic marker protein. For Rab27B these data are consistent with a prior report demonstrating Rab3 and Rab27B GTPases colocalize to presynaptic elements in cultured hippocampal neurons (Pavlos & Jahn, 2011), although the GTPases were observed to differentially distribute among SV, with only a small population of SV’s demonstrating enrichment of both GTPases. Our analysis on EM micrographs of hippocampal synapses from WT and Rab27B KO mice demonstrated that Rab27B KO resulted in a slight increase in the diameter of presynaptic SV. This result differs from that of Rab27B KO in pancreatic acinar cells where a reduction in zymogen granule diameter relative to WT controls was reported (Hou et al., 2016). The change in diameter may result as Rab proteins participate in vesicle biogenesis from donor membrane compartments through multi‐subunit Rab tether complexes, regulatory complexes that interact with coat proteins, and that influence vesicle budding (Angers & Merz, 2011). In spite of the increase in SV diameter, our data indicate this did not noticeably alter the amount of neurotransmitter packed per SV, as the amplitude of the mEPSC was the same in WT and KO mice. These effects appear unique to Rab27B, as Rab3A KO was reported to be without effect on SV diameter or on frequency of spontaneous neurotransmitter exocytosis (Schluter et al., 2004).

The actions of Rab27B on evoked synaptic transmission differed considerably between the SC‐CA1 and DG/MF‐CA3 regions and reflect in specific aspects that previously reported for Rab3A KO mice. Input–output relationships were unchanged from control at the SC‐CA1 synapse similar to that reported for Rab3A KO, but significantly increased at the DG/MF‐CA3 synaptic region, a result differing from a prior report showing no change in the Rab3A KO (Castillo et al., 1997; Geppert et al., 1994). A lack of effect of Rab27B KO on paired pulse at either the SC‐CA1 or DG/MF‐CA3 synaptic regions reflects similarity to that observed in the Rab3A KO. Notably, while frequency facilitation was increased in both Rab27B and Rab3A KO slices at the SC‐CA1 synapse (Geppert et al., 1994), we report here that KO of Rab27B results in a significant decrease in 14 Hz frequency facilitation, which also trends across the frequencies tested. These data demonstrate that Rab27B likely acts uniquely different at these synaptic regions throughout the frequency range tested. That is, in SC‐CA1 synapses, deletion of Rab27B resulted in SC’s resistant to synaptic depression without, based on our paired pulse data, affecting the probability of neurotransmitter release. One possibility is that lack of Rab27B at the SC pre‐synapse increases the refilling rate of the readily releasable pool to sustain evoked release during moderate activity (≤14 Hz). By comparison, DG/MF‐CA3 synapses displayed a ~50% reduction in the magnitude of frequency facilitation compared to WT controls. These effects are different from what has been reported for Rab3A knockout mice, where 14 Hz stimulation caused synaptic depression in SC (Geppert et al., 1994), and MF‐CA3 synapses showed equivalent responses to WT mice (Castillo et al., 1997). Thus, although Rab27B and Rab3A have been reported to share many identified effectors and based on sequence homology suggested to act in a complementary and redundant fashion, our data strongly suggest certain overlapping but also clearly independent synaptic actions, as the absence of one of the Rab GTPases, in the continued presence of the other GTPase, can exert substantive and unique functional effects on frequency facilitation.

A central form of synaptic plasticity identified in synaptic information storage, and that is thought to underly learning and memory, is LTP (Bliss, Collingridge, & Morris, 2003; Lynch, 2004). As such, establishing the molecular mechanisms that govern LTP is key to understanding information storage in neural circuits. The hippocampal region is critical for the acquisition of episodic memory, with DG/MF‐CA3 and SC‐CA1 synapses expressing, respectively, predominantly NMDA receptor‐independent (Harris & Cotman, 1986; Nicoll & Schmitz, 2005) and NMDA receptor–dependent early phase LTP (Collingridge, Kehl, & McLennan, 1983; Harris, Ganong, & Cotman, 1984; Morris, Anderson, Lynch, & Baudry, 1986). Here we demonstrate that complete deficiency of Rab27B expression exerts no effect on electrical activity induction of LTP at SC‐CA1 hippocampal synapses but results in complete elimination LTP induction at DG/MF‐CA3 synapses. The loss of LTP at the DG/MF‐CA3 synapse in the Rab27B KO occurred in spite of no difference in the amplitude of activity evoked baseline EPSPs or PPR relative to WT control hippocampal slices. These data suggest that contributions of presynaptic calcium to synaptic transmission and plasticity were likely not accountable for Rab27B KO and WT synaptic differences. This is consistent with a report showing no reduction in inward calcium current following anti‐Rab27 antibody injection into the pre‐terminal of the squid giant synapse (Yu et al., 2008). Moreover the deficit in activity induction of LTP at the DG/MF‐CA3 synapse was rescued by viral‐mediated expression of the human Rab27B protein in the KO condition. While the molecular mechanism underlying the essential action of Rab27B in LTP at the DG/MF‐CA3 synapse remains unknown, it is important to note that Rab3A KO exhibits an identical loss of activity‐induced LTP at DG/MF‐CA3, but not SC‐CA1 synaptic regions (Castillo et al., 1997; Geppert et al., 1994).

The presynaptic action of Rab27B KO on LTP within DG/MF‐CA3 pathway is likely related to the central role that Rab‐proteins exert in virtually all steps of vesicular trafficking, including vesicular transport along cytoskeletal elements, vesicular tethering and fusion to the target membrane (Hutagalung & Novick, 2011). One known effector of Rab3 is RIM1 which has been reported to form a trimeric complex containing Rab3 and Munc13 (Castillo et al., 2002; Schoch et al., 2002), a protein essential for exocytotic activity in mammalian glutaminergic neurons. The KO of Rab27B exhibited negligible effect on expression of the array of synaptic proteins that were assessed, with the exception of tomosyn‐1, a v‐SNARE containing protein that serves as a negative regulator of exocytosis (Hatsuzawa et al., 2003; Yizhar et al., 2004) and regulator of SV distribution between functionally defined resting and recycling SV pools in hippocampal neurons (Cazares et al., 2016). Tomosyn‐1 also serves as an effector of Rab3, and perhaps Rab27, GTPases in regulating SV pool dynamics (Cazares et al., 2016). Moreover knockdown of Tomosyn‐1 at MF‐CA3 synapses by shRNA strongly impairs MF‐CA3 frequency facilitation and LTP (Ben‐Simon et al., 2015). This raises the possibility that the reduction in Tomosyn‐1 occurring on Rab27B KO may have been a contributor to effects we observed at the DG/MF‐CA3 synapse, although this may not exclude requirement of sequential or essential actions of both Rab27 and Rab3 GTPases. The role of a decrease in tomosyn expression in the Rab27B KO is, unlikely to support the observed increase in frequency of mEPSC, as KO of tomosyn‐1 has been reported to have no effect on spontaneous membrane fusion (Yizhar et al., 2004). In addition to Rab GTPase actions on SV trafficking, anterograde transport of the brain‐derived neurotrophic factor (BDNF) TrkB receptors to the surface of axon terminals, is mediated by a Slp1/Rab27B/CRMP‐2 complex, which directly links TrkB to kinesin‐1 (Arimura et al., 2009). The role of BDNF in LTP has been extensively studied in the hippocampus as well as other brain regions, with BDNF exerting actions at both pre‐ and post‐synaptic elements (Leal, Afonso, Salazar, & Duarte, 2015).

The CA3 circuits of the hippocampus exert key actions in early stages of memory acquisition, with CA3 principal cells (PCs) receiving excitatory inputs from recurrent CA3 collaterals (associational‐commissural fibers (A‐C)), entorhinal cortex projections and MF inputs from DG granule cells. The different inputs vary considerably in their structural and functional properties (Rebola, Carta, & Mulle, 2017), with A‐C fibers within the stratum radiatum requiring NMDA receptor activation for LTP. Behaviorally, MF synapses are believed required for the acquisition of contextual memories (Rolls, 2013), which suggests an importance to testing for a functional action of the Rab27B KO on such memory acquisition in future studies. In addition, as CA3 subfields demonstrate NMDA receptor‐dependent and independent forms of LTP, this hippocampal region may serve valuable to further define within the same CA3 hippocampal region that Rab27B actions are NMDA‐receptor independent, as demonstrated here for the DG/MF‐CA3 pathway. In the current studies, sensitivity of the electrically stimulated EPSP to the metabotropic glutamate receptor agonist (2S,1’R,2’R,3’R)‐2‐(2,3‐dicarboxycyclopropyl)glycine (DCG‐IV, 3 mM) was tested in each experiment to confirm MF stimulation (Yoshino, Sawada, Yamamoto, & Kamiya, 1996).

In summary, the results presented here provide a novel perspective on the role of the presynaptic exocytotic Rab GTPases Rab 27B, and of the Rab3 subfamily. That is, Rab27B KO results in similar actions on LTP as observed in the Rab3A KO, in spite of Rab3A remaining present in the Rab27B KO. In addition, the Rab27B KO resulted in a divergence in effects on high frequency facilitation at SC‐CA1 and MF‐CA3 synaptic sites, likely resulting from site‐specific interactions that depend on the presence of particular signaling effectors or membrane receptors. Our data further indicate that both functional Rab27B and Rab3A are required for LTP induction within the DG/MF‐CA3 region of glutamatergic synaptic terminals in the hippocampus. We propose that Rab27B and Rab3A act synergistically perhaps via sequential effector recruitment or signaling for presynaptic LTP expression in this region. Unraveling the precise molecular mechanisms governing the presynaptic actions of Rab3 and Rab27B GTPases will be central to identifying this synapse specificity.

## CONFLICT OF INTEREST

The authors declare no competing financial interests.
